# Antagonistic interactions between honey bee bacterial symbionts and implications for disease

**DOI:** 10.1186/1472-6785-6-4

**Published:** 2006-03-21

**Authors:** Jay D Evans, Tamieka-Nicole Armstrong

**Affiliations:** 1 USDA-ARS Bee Research Lab, BARC-East Bldg. 476, Beltsville, MD 20705, USA

## Abstract

**Background:**

Honey bees, *Apis mellifera*, face many parasites and pathogens and consequently rely on a diverse set of individual and group-level defenses to prevent disease. One route by which honey bees and other insects might combat disease is through the shielding effects of their microbial symbionts. Bees carry a diverse assemblage of bacteria, very few of which appear to be pathogenic. Here we explore the inhibitory effects of these resident bacteria against the primary bacterial pathogen of honey bees, *Paenibacillus larvae*.

**Results:**

Here we isolate, culture, and describe by 16S rRNA and protein-coding gene sequences 61 bacterial isolates from honey bee larvae, reflecting a total of 43 distinct bacterial taxa. We culture these bacteria alongside the primary larval pathogen of honey bees, *Paenibacillus larvae*, and show that many of these isolates severely inhibit the growth of this pathogen. Accordingly, symbiotic bacteria including those described here are plausible natural antagonists toward this widespread pathogen.

**Conclusion:**

The results suggest a tradeoff in social insect colonies between the maintenance of potentially beneficial bacterial symbionts and deterrence at the individual and colony level of pathogenic species. They also provide a novel mechanism for recently described social components behind disease resistance in insect colonies, and point toward a potential control strategy for an important bee disease.

## Background

Insects, like many eukaryotes, can be strongly affected by the microbes they harbor. Bacterial associates of insects are implicated in the degradation of plant materials and other foods, regulation of pH, synthesis of vitamins and, in relatively rare cases, induction of disease [1]. Insect-bacteria associations range from facultative short-term interactions to highly codependent symbioses [2]. Social insects provide unique resources for microbial symbionts, thanks to the high density of individuals within colonies, sharing of food and other resources, and the coexistence of colony members from multiple generations. Not surprisingly then, symbioses between social insect species and microbial species are common and often highly coevolved. Many species of termites and ants, for example, are obligately tied to specific microbes for their nutritional needs [3-6].

Honey bees, *Apis mellifera*, support a diverse microbial biome [7]. Recent surveys from adult bees indicate the presence of dozens of bacterial taxa, ranging from gram-positive bacteria to alpha-, beta-, and gamma-proteobacteria [7,8]. While a few of these bacterial species are clearly pathogenic, most have never been associated with honey bee disease and their impacts on each other and their honey bee hosts are unknown.

Now is an ideal time to explore the diversity of insect endosymbiotic bacteria. First, an immense library of sequence data for 16S rRNA loci and other robust markers allows the precise identification of many associated species, even those that resist cultivation [9]. Over 200,000 bacterial entries exist currently for 16S rRNA, and 16S sequences can place most surveyed bacterial taxa securely into genera, if not species. Additionally, genomic studies of diverse bacterial species allow new insights into the mechanisms of maintenance and growth for these microbes as well as their potential impacts on the health of their insect hosts [10-12]. Finally, recent community-level surveys of bacterial diversity across different arthropods [13-18] allow a comparative approach toward understanding roles played by bacteria during different host life stages, and in different organs of the body.

Recently, we described four honey bee bacterial symbionts with clear bacteriostatic effects against the most virulent and widespread honey bee pathogen, the gram-positive bacterium *Paenibacillus larvae larvae *[19]. Here, we present a more complete survey of bacterial species isolated from honey bee larvae: the life stage at which they are most susceptible to invasion by pathogenic bacteria. We used 16S universal bacterial primers to identify bacteria and *in vitro *inhibition assays to quantify the abilities of each of these isolates to inhibit *P. l. larvae*. We present wide-ranging taxa capable of inhibiting this pathogen and show considerable variation within and across colonies in the distribution of inhibitory taxa. The results have general implications for the expression of bacterial virulence in insects and for the maintenance of both beneficial and disease-associated bacteria in social insects. They also point to new avenues for the prophylactic or therapeutic treatment of honey bee diseases.

## Results and discussion

### Species distribution

Sequenced isolates (n = 61) were placed by 16S rRNA sequence similarity into four bacterial genera: *Acinetobacter, Bacillus, Brevibacillus*, and *Stenotrophomonas*. A total of 43 16S haplotypes representing a minimum of 15 bacterial taxa were identified from the survey (Table 1, Fig. 2). Most isolates fell within the genus *Bacillus *(n = 41). Of these, 29 belonged to the *B. cereus *group and differed only slightly at 16S. While 16S rRNA fails to confidently resolve this species group, 26 showed best matches to *B. cereus s.s. isolates*, while three were closest to *B. thuringiensis*. Additional resolution of this group was provided by GLyP and PyC sequencing for a subset of isolates. These sequences reflected a broad range of haplotypes with matches to members on each extreme of the *cereus *group (Fig. 3). Outside of the *cereus *group, the remaining *Bacillus *spp. matched *B. fusiformis*, *B. flexus*, *B. mycoides*, and *B. niabensis *(Table 1). Three additional *Bacillus *isolates showed <97% sequences similarity to deposited sequences in GenBank and as such were not reliably assignable to species. However two of those three isolates fell in a clade with *B. fusiformis *and the last isolate extended off the *B. cereus *clade (Fig 2). 10 isolates were indistinguishable from *Stenotrophomonas maltophilia *(Table 1). Although all three isolates of *Acinetobacter *fell into one clade in the 16S tree, BLASTN only matched one of the three isolates to a species while the remaining were matched solely to a genus. Seven isolates were placed into the genus *Brevibacillus*, with close matches to *Br. formosus *(n = 4), *Br. centrosporus *(n = 1), and *Br. brevis *(n = 1). The final *Brevibacillus *isolate did not match particularly closely to a taxon in the 16S database. None of the genera represented in this survey matched genera found in a previous 16S survey of bacteria from adult honey bees [8], suggesting that bacterial sequencing in bees will continue to identify novel taxa.

### Diversity of inhibitory species

Inhibition zones, when present, ranged from a <10 mM radius around the isolated bacteria to complete inhibition of *P. larvae *across the dish. A total of 23 bacterial isolates consistently inhibited *P. larvae*. Isolates that inhibited *P. larvae *were evenly distributed across the sampled taxa (Fig. 2). Out of 43 *Bacillus sp*. isolates, ten showed consistent inhibition while seven additional isolates showed conditional inhibition. These seven all fell into the *Bacillus cereus *group. Of the ten *Bacillus *isolates with consistent inhibition, 8 were *B. cereus *and two were *B. fusiformis*. Thus, by the current *in vitro *assay, *B. niabensis, B. circulans, B. flexus*, and *B. mycoides *isolates were not inhibitory of *P. larvae*. All isolates that were matches with *Brevibacillus formosus *consistently inhibited the pathogen, while a single isolate placed with *B. centrosporus *did not inhibit. All isolates tied to *Stenotrophomonas maltophilia *consistently inhibited *P. larvae*, while two of three *Acinetobacter *isolates showed inhibition.

### Distribution across individual bees and colonies

More bacteria and bacterial species were isolated, per individual, from 7-day-old larvae than from one-day larvae (28 of 55 larvae at 7 days, 28 of 306 larvae at 24 hr, G test, p < 0.0001; Table 2). Since larvae for both incubation lengths were collected at the same time from the colony, this difference does not reflect a greater chance for larvae to be inoculated in the nest as they age. Instead, the differences presumably reflect quantitatively higher bacterial loads in older individuals, such that these bacteria were more readily cultured. There was no apparent difference in species type or overall diversity between young and older larvae, nor was there a significant difference in the likelihood that a collected bacterial species was inhibitory toward *P. larvae *(likelihood ratio, p = 0.2).

There was a significant difference across sites in the frequency of resident, cultivable, bacterial species. Two sites had very low bacterial levels. One of these sites had been established shortly before collections took place. In this site (LID), only 4 out of 136 larvae (from 4 out of 34 colonies) showed bacterial growth. A second site, (Poultry) showed similarly low rates of cultivable bacteria (2.2%, n = 45 larvae). By contrast, measurable bacterial loads were present in 13% (n = 63) and 24% (n = 46) of larvae from the Parasitology and Meadow sites, respectively. Within sites, there was significant variation across colonies in the tendency of their larvae to harbor bacteria (nested ANOVA, p < 0.001), and in the specific bacteria found in certain colonies. More widespread surveys will be useful for defining colony-level bacterial 'signatures', an important step in determining the impacts of symbiotic bacteria on colony health.

## Conclusion

We describe wide-ranging endogenous bacterial taxa that are capable of inhibiting an important honey bee pathogen and show considerable variation within and across colonies in the distribution of these taxa. The results have general implications for the expression of bacterial virulence in insects and for the maintenance of both beneficial and disease-associated bacteria in social insects. They also point to new avenues for the prophylactic or therapeutic treatment of honey bee diseases. None of the genera represented in this survey matched genera found in a previous 16S survey of bacteria from adult honey bees [8], although they do mimic, broadly, the microbial biome measured in bee colonies to date (as reviewed by Gilliam, [7]).

Most of the bacteria cultivated in this study belonged to the genus *Bacillus*, a result that is consistent with the high frequency of isolates placed in this genus by Gilliam and colleagues [7]. Among the *Bacillus *species, the majority fell into the *Bacillus cereus *group. Both 16S rRNA sequencing and multi-locus sequencing (GlyP, PyC) indicate that these isolates represent several distinct taxa from this group, although interference with *P. larvae *did not fall out with species identification. The high frequency of bees harboring bacteria from the *B. cereus *group suggests a stable symbiosis between bees and this taxon, perhaps helping to explain the fact that bees are more tolerant than many other insects toward *B. thuringiensis *[20]. Curiously, isolates from this group which shared both 16S haplotypes and sequence identity at the two protein-coding genes differ substantially in their ability to inhibit *P. larvae*. This variation could result from undetected genetic variation within subspecies, conditional activation of inhibitory substances, or a role for plasmids or other mobile elements in inhibition. Future experiments will help resolve the causes of conditional inhibition by *Bacillus cereus *subspecies.

There is a growing appreciation for the potentially beneficial roles of bacteria in honey bee colonies. Evans and Lopez [21] recently showed that non-pathogenic bacteria can stimulate the innate immune response of honey bee larvae, perhaps helping bees survive exposure to pathogens. Further, Reynaldi et al. [22] recently showed that bacteria isolated from bees in Argentina are inhibitory of the important bee fungal pathogen, *Ascosphaera apis*. It will be interesting to determine whether these species, in addition, are also inhibitory toward *P. larvae*, and to contrast the microbes associated with bees across different continents.

Bacterial symbionts likely play roles in individual and colony fitness across the social insects. Sharing of symbiotic bacteria is notoriously important for termite nutrition, and it is increasingly clear that both obligate and facultative symbioses are widespread in social insects. Recent evidence for a socially communicable defense against pathogens in termites [23] might indeed reflect sharing of bacteria among termite colony members, rather than the proposed induction of host-specific physiological changes.

Perhaps, as is apparent in the termites and ants [24,25], honey bees have evolved behavioral or physiological mechanisms to enhance the transmission of beneficial microbes, while battling those species which are pathogenic. This would indicate a delicate balancing act for bees and other social insects, allowing for the encouragement of beneficial species while maintaining barriers against exploitation by pathogens. If so, discrimination at the levels of behavior and individual immune responses might be used to bias the microbial biome within insect colonies toward mutualists and against parasites and pathogens. Beneficial symbionts can potentially be fed to developing bees as a prophylactic against disease [21], and can regardless be used to better understand the complexity of interactions between the microbial biota of bees. It will, in this vein, also be important to look more closely at transmission mechanisms of microbes within and between bee colonies.

## Methods

### Organisms

**Figure 1:**
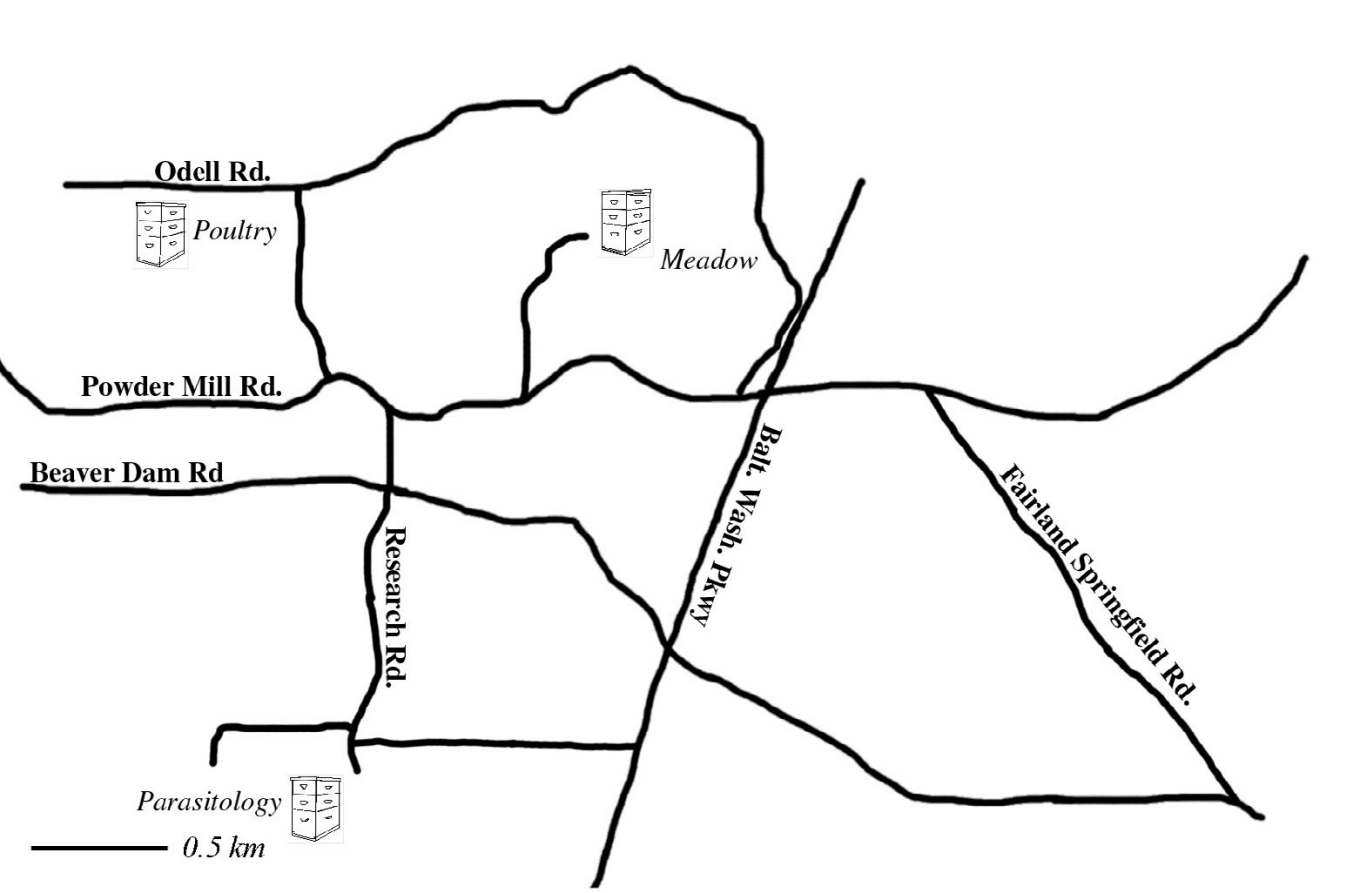
**Apiary Map**. Map of the apiaries in which honey bee larvae were collected. Q3, Q7, and LID larvae resided in colonies within the Meadow apiary. Size bar represents 0.5 km.

**Figure 2:**
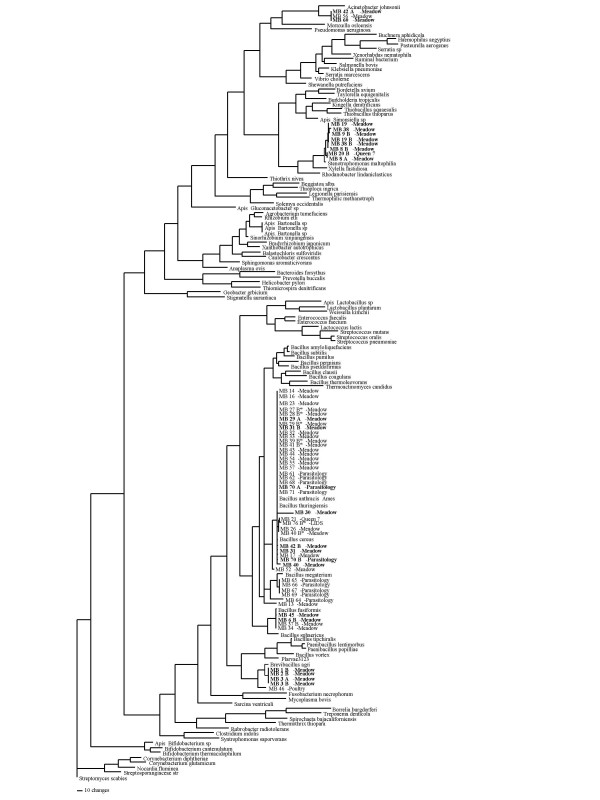
**16S rRNA parsimony tree**. Maximum parsimony tree showing relationships between bacterial isolates from this study, other bacteria known from bees, and representative members of the major bacterial clades. Tree based on an alignment of 850 nt from the 5' end of the 16S rRNA gene. Bacteria from this study shown as MB##, followed by site of collection. Isolates that showed inhibition of the honey bee pathogen *P. larvae *shown in bold type. Names in bold represent those isolates that inhibited and isolates followed by an asterick did not inhibit consistently.

**Figure 3:**
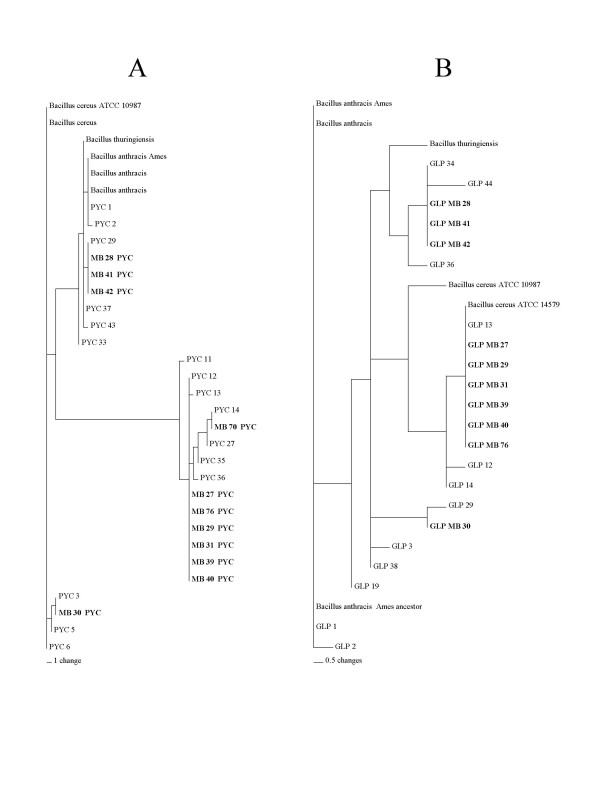
**MLST sequence analysis trees**. Maximum parsimony trees representing the B. cereus group, including types for *B. cereus, B. anthracis*, and *B. thuringiensis *and representatives from [29]. Relationships were established using two of the six primers generally used to distinguish the three *Bacillus *type bacteria within this group (GlyP, and PyC; [29]. Alignments generated from a 520 bp alignment of the GLP locus and a 520 bp alignment of the PYC locus. Isolates from honey bees shown in bold.

Bacteria were cultivated from a total of 341 honey bee larvae collected from four apiaries near the USDA-ARS Bee Research Laboratory, Beltsville, MD, USA from June-August, 2003 (Fig. 1). These larvae were collected from both mature honey bee colonies (n = 217 larvae in 51 colonies established at least one year prior to collection) and from colonies that had been newly established (134 larvae from 34 colonies). First-instar larvae were collected and reared for 24 h or 7d using an aseptic artificial diet, controlled temperature (34°C) and high humidity as described [19]. Larvae were frozen at -80°C prior to the cultivation and isolation of bacteria.

### Inhibition assays

Individual larval bees were ground in 40 ul sterile H_2_O at room temperature, using a disposable pestle. A filter-paper disk was impregnated with 20 ul from the resulting suspension. Each disk was centered on a standard Petri plate (100 × 15 mm) consisting of Brain-Heart Infusion (Difco) agar media containing 0.1 mg/ml thiamine hydrochloride as described [26]. Prior to placement of these disks, plates had been inoculated with a lawn of approximately 1 × 10^8 ^viable spores of *P. larvae*. These spores were isolated from diseased honey bee larvae collected in 2002 from a single bee colony in Berkeley, CA, U.S.A (BRL sample 230010; [25]). After 24 h incubation at 34°C, plates were scored for both bacterial growth and inhibition toward *P. larvae*. Bacterial growth was described as any bacteria on or contiguous to the paper disks that was atypical for *P. larvae*. Inhibition was defined as the radial distance between these paper disks and the first line of *P. larvae *growth. *P. larvae *inhibition was only observed in conjunction with growth of larval-derived bacteria (e.g., there were no signs of inhibition resulting from the larvae themselves by this assay). Inhibition was confirmed in all cases by replating collected bacterial cultures against a fresh lawn of *P. larvae*.

### DNA extraction and sequencing

All bacterial colonies on or alongside paper disks were collected with a sterile wand. Approximately 10 mg vegetative cells of each sample was suspended in 300 ul of 30% glycerol solution then kept at -20°C. To isolate DNA, 50 ul from this suspension was mixed with 50 ul 10% Chelex-100 (Bio-Rad, Hercules, CA), then incubated at 72°C for 10–20 minutes before being placed on ice.

16S rRNA genes were amplified by PCR using universal eubacterial primers eu27.F and eu1495.R [27]; sequences 5'- gagagtttgatcctggctcag-3' and 5'- ctacggctaccttgttacga-3', respectively). Reaction mixes included 2 ul bacterial extract, 2 U Taq DNA polymerase with recommended buffer (Boehringer, Indianapolis, IN), 1 mM DNTP mix, 2 mM MgCl_2_, and 0.2 μM of each primer. PCR was carried out on an MJ-Research PTC-100 thermal cycler using 30 cycles of 93°C 1 min, 54°C 1 min, 72°C 1 min. Bands of an appropriate size were confirmed by agarose gel electrophoresis, then PCR products were purified directly (Gene Pure). Products were sequenced using Big Dye 2.0 (Applied Biosystems, Carlsbad, CA) end-terminal cycle sequencing, followed by separation and analysis on an Applied Biosystems 3130 DNA Analysis machine. Sequencing was carried out in one direction from the 5' (eu27.F) end.

### Sequence analyses

Sequences were checked and trimmed using the software program Sequencher (Gene Codes), then were aligned using the CLUSTALW algorithm, invoked by Omiga 2.0 (Oxford Molecular). Alignments also included a diverse array of bacterial species, including all of those previously identified from bees and representative members of the major bacterial clades. Alignments were exported to PAUP 4.0b10 (Sinauer) to generate phylogenetic hypotheses using a heuristic maximum-parsimony algorithm. Trees were generated for the entire data set, and for a data set limited to species found in the genus *Bacillus*. Sequences were also compared directly to all 16S rRNA sequences deposited in GenBank [28] using BLASTN, in August, 2005.

### Multilocus sequences for Bacillus isolates

A large fraction of sequenced isolates were placed into the *Bacillus cereus *group by 16S rRNA similarity. To better resolve members of this group, isolates were sequenced at two protein-coding loci that offer informative sequence variation within this group [29]. Glycerol uptake factor protein (PCR and sequencing primers Glyp.F GCG TTT GTG CTG GTG TAA GT and GlyP.R CTG CAA TCG GAA GGA AGA AG) and Pyruvate carboxylase (primers PyC.F GCG TTA GGT GGA AAC GAA AG and PyC.R CGC GTC CAA GTT TAT GGA AT) genes were amplified by a three-step PCR regime of (94 30 s, 58 30 s, 72 1 m) × 30 and sequenced via Big-Dye N terminal sequencing as described above. Sequences were aligned with each other and voucher sequences from the Multilocus sequencing database  using CLUSTAL, then were analysed by maximum parsimony using PAUP 4.0b.

## Authors' contributions

JDE conceived of the project and both authors were involved with data collection, analysis, and writing.

## Figures and Tables

**Table 1:** Isolate identification, 16SrRNA best match, similarity and BLASTN e-value, and radial zone of inhibition against the bee pathogen *Paenibacillus l. larvae*.

MB ID	**Genbank ID**	**Best Match**	**% Similarity**	**e-value**	Inhib.
BRL02-1	DQ339635	*Brevibacillus formosus*	98	0	**23**
BRL02-2	DQ339636	*Brevibacillus formosus*	98	0	**13**
BRL02-3A	DQ339637	*Brevibacillus brevis*	97	0	**9**
BRL02-3B	DQ339638	*Brevibacillus formosus*	98	0	**>40**
BRL02-4	DQ339639	*Stenotrophomonas maltophilia*	99	0	**0**
BRL02-5	DQ339640	*Stenotrophomonas maltophilia*	88	0	**0**
BRL02-6A	DQ339641	*Bacillus fusiformis*	98	0	**>40**
BRL02-6B	DQ339642	*Stenotrophomonas maltophilia*	99	0	**>40**
BRL02-7	DQ339643	*Stenotrophomonas maltophilia*	97	0	**0**
BRL02-8	DQ339644	*Stenotrophomonas maltophilia*	99	0	**>40**
BRL02-9	DQ339645	*Stenotrophomonas maltophilia*	98	0	**>40**
BRL02-11	DQ339646	*Stenotrophomonas maltophilia*	88	-179	**0**
BRL02-13	DQ339647	*Bacillus niabensis*	99	0	**0**
BRL02-14	DQ339648	*Bacillus cereus*	99	0	**0**
BRL02-16	DQ339649	*Bacillus cereus*	98	0	**0**
BRL02-17	DQ339650	*Bacillus cereus*	99	0	**0**
BRL02-19	DQ339651	*Stenotrophomonas maltophilia*	99	0	**18**
BRL02-20	DQ339652	*Stenotrophomonas maltophilia*	99	0	**35**
BRL02-21	DQ339653	*Bacillus cereus*	99	0	**0**
BRL02-22	DQ339654	*Bacillus cereus*	99	0	**0**
BRL02-23	DQ339655	*Bacillus cereus*	99	0	**0**
BRL02-24	DQ339656	*Brevibacillus centrosporus*	79	2E-90	**0**
BRL02-25	DQ339657	*Bacillus cereus*	98	0	**0**
BRL02-26	DQ339658	*Bacillus cereus*	99	0	**0**
BRL02-27	DQ339659	*Bacillus cereus*	100	0	**A**
BRL02-28	DQ339660	*Bacillus cereus*	99	0	**A**
BRL02-29	DQ339661	*Bacillus cereus*	99	0	**0**
BRL02-30	DQ339662	*Bacillus cereus*	91	0	**0**
BRL02-31	DQ339663	*Bacillus cereus*	99	0	**12**
BRL02-32	DQ339664	*Bacillus cereus*	99	0	**0**
BRL02-33	DQ339665	*Bacillus cereus*	98	0	**0**
BRL02-34	DQ339666	*Bacillus fusiformis*	99	0	**0**
BRL02-37	DQ339667	*Bacillus fusiformis*	98	0	**>40**
BRL02-38	DQ339668	*Stenotrophomonas maltophilia*	99	0	**>40**
BRL02-39	DQ339669	*Bacillus cereus*	99	0	**0**
BRL02-40	DQ339670	*Bacillus cereus*	99	0	**0**
BRL02-41	DQ339671	*Bacillus cereus*	99	0	**0**
BRL02-42A	DQ339672	*Acinetobacter calcoaceticus*	99	0	**>40**
BRL02-42B	DQ339673	*Bacillus cereus*	99	0	**>40**
BRL02-43	DQ339674	*Bacillus cereus*	98	0	**0**
BRL02-44	DQ339675	*Bacillus cereus*	99	0	**0**
BRL02-45	DQ339676	*Bacillus fusiformis*	97	0	**>40**
BRL02-46	DQ339677	*Brevibacillus centrosporus*	97	0	**0**
BRL02-52	DQ339678	*Bacillus mycoides*	98	0	**0**
BRL02-54	DQ339679	*Bacillus cereus*	99	0	**0**
BRL02-55	DQ339680	*Bacillus cereus*	99	0	**0**
BRL02-56	DQ339681	*Acinetobacter sp*.	100	0	**0**
BRL02-57	DQ339682	*Bacillus cereus*	100	0	**0**
BRL02-60	DQ339683	*Acinetobacter sp*.	100	0	**>40**
BRL02-61	DQ339684	*Bacillus cereus*	98	0	**0**
BRL02-62	DQ339685	*Bacillus cereus*	99	0	**0**
BRL02-64	DQ339686	*Bacillus circulans*	96	0	**0**
BRL02-65	DQ339687	*Bacillus flexus*	99	0	**0**
BRL02-66	DQ339688	*Bacillus flexus*	99	0	**0**
BRL02-67	DQ339689	*Bacillus flexus*	99	0	**0**
BRL02-68	DQ339690	*Bacillus cereus*	100	0	**0**
BRL02-69	DQ339691	*Bacillus flexus*	100	0	**0**
BRL02-70	DQ339692	*Bacillus cereus*	99	0	**18**
BRL02-71	DQ339693	*Bacillus cereus*	100	0	**0**
**BRL02-76**	DQ339694	***Bacillus cereus ***	**97**	**0**	**8**
